# Scraps

**Published:** 1901-09

**Authors:** 


### SCRAPS

PICKED UP BY L. M. G.

**Clinical Records** (36).—Mr. William Hughes, one of the joint managers of the Prudential Assurance Company, in a paper on the medical examination for life assurance read before the Newcastle Insurance Institute, says, referring to the family history : In many cases applicants are really ignorant of the facts of their family history, or at most can only state that their parents and so many of their brothers and sisters are dead. In such a case an examiner will sometimes accept the convenient answer, "causes of death unknown," but the office is seldom content to accept so vague an answer and will press for further information. More often causes will be given at random, "some kind of fever," "a severe cold," or some equally vague statement being made. In one case that came under my notice an applicant stated that he could not say exactly what his mother died of, but he knew it was "nothing serious!"—*The Medical Examiner.*

**Modern Fashions.**—First lady : "I'm taking four kinds of medicine. How many are you taking?" Second lady : "Oh, medicine don't count. Operations are all the go now. I've had three this summer."

This little story came from St. Louis long before the Cheltenham meeting of the British Medical Association, but it is only another way of putting the matter to which Dr. Goodhart referred when, in the "Address in Medicine,"

he said: "Men and women in the present day rush into the not always sufficiently repellent arms of surgery. A little pain unnerves them, and all they know of surgery is its successful side. . . . Who does not know the difficulty there is in preventing people from undergoing a serious operation for the purpose of stitching their harmless mobilities—for it is only quite exceptional that it is otherwise—into their places? It is the same in many another region, throats and noses suffer terribly from this lust of operation that has beset the public."

**The Debt of the Public to the Medical Profession.**—In Mr. John Wanamaker's gallery is one of the most striking pictures I have ever seen. On a large canvas by Fritel, in the centre of the picture, advancing directly toward the spectator, is a large cavalcade of warriors arrayed in corselet and casque. Their stately march at once arrests the eye. The leader is Julius Cæsar. He is flanked by Napoleon and Alexander the Great, and followed by Attila, Semiramis, and a lengthening host of those whom the world counts among its greatest conquerors. They advance between two long rows of rigid, ghastly corpses, all stretched at right angles to their line of march. Spectral mountains in the distance hedge in a desolate plain given over to the vulture, the bat, and silence.

I would that some artist might paint a companion picture of the "conquerors in medicine" instead of the "conquerors in war." Instead of spectral hills and a barren waste, the scene should be laid in a fertile, smiling valley, bounded by delectable mountains. The stately procession should be led by Edward Jenner. He should be flanked by Joseph Lister and John C. Warren, and followed by Simpson, Billroth, Livingstone, Ambroise Paré, Virchow, John Hunter, and many a modest but unknown hero who has yielded up his spirit in the performance of his duty. Instead of treading their way between lines of corpses, they should march between lines of grateful men and women and a host of God's little children who, on bended knee and with clasped hands, would reverently invoke Heaven's richest benediction upon their deliverers.

Thus should humanity recognise its debt to the medical profession.—Dr. W. W. Keen, the *Philadelphia Medical Journal.*

**Missions in China.**—At Fuh-Ning the medical work was in the hands of the Rev. Dr. Synge, Dr. Mary Synge, and the Rev. Dr. Mackenzie. The last-named has not a high opinion of the native doctors, indeed, he believes that the mere ceasing to take the remedies which they prescribe would often suffice to restore men to health. He gives one or two instances of the treatment in vogue:—

In November I attended a little boy in Fuh-Chow suffering from a severe attack of pneumonia; the native doctor when called said the trouble was unimportant, but his mother was to give him medicine, viz., to make a sort of tea of (what I saw bought) a few wizened-up prunes, pieces of bamboo tree, grains of wheat. Next week, in Lo-Ngwong Mission station compound, I saw a woman suffering from ophthalmia; her eyes, by advice, were caked with some brownish powder, and a piece of "silver paper" filled up the inner corner of one eye.

The Rev. L. H. F. Star, of the well-known Bristol family, was in general charge of the Mission at Fuh-Ning. Dr. Main says of the people at Hang-Chow:—

They smell of disease, poverty, and misery. They are unsavoury and insanitary, and are covered with disease from head to heel, and also covered with some of the lower forms of insect life which do not bear a high reputation! China is still fast asleep on the subject of sanitary improvements and personal cleanliness. The one who can turn away such without trying his level best to help them by word and deed is not worthy of the work to which he has been called. No urgent case was refused admittance, but less serious ones were encouraged to seek relief in the out-patient department.

Our difficulties in connexion with the treatment of in-patients, although very much less than they used to be, are still often very great and trying, and tax our patience to its utmost limit. Chinese nurses lack will power, and find great difficulty in dealing with those who belong to a higher social rank than themselves, but are sadly wanting in ideas of cleanliness.

The difficulty of dieting special cases is very real, and the patients are ever getting their friends to smuggle in all kinds of dainty dishes, from putrefying bean curd to pork dumplings, turnips, and peanuts. There was one dietetic [*sic*] patient whom we were dieting on pork, port wine, almond emulsion, brown bread, &c., &c., at an average cost of $2 per diem, but no rice was allowed him, as he was told it was bad for his disease. However, his zeal for rice exceeded his knowledge and our advice, and in the small hours of the morning, when everyone was asleep, he went to the kitchen and disposed of large quantities of the cold article! He baffled all our plans, and finally we had to send him home, where he shortly afterwards died. It is not uncommon to find a patient with acute dysentery or typhoid fever refreshing himself with a water nut or a Chinese pear, the hardness of which is only known to those who have tried to eat a piece of stick dipped in syrup.

The demoralization and corruption caused by opium smoking can hardly be overstated. The passion for opium corrodes the soul of the Chinese. What principles they have are cast to the winds, and all regard for truth, purity, and duty are crushed out of their hearts. The love of opium is with them the root of all evil. Surely we ought to adopt means and measures to alleviate and finally root out this shameful evil, which is a disgrace to our English name. Without this I have no hesitation in saying that the recovery of the country, whether physically, morally, or mentally, is an impossibility. With the advent of new reforms and new forces of civilization, and the spread of Western learning and Christianity a powerful influence can be exerted in favour of winning the Chinese from the degrading vice.—*Church Missionary Society's Report.*

**Medical Philology** (XXXIX.).—In his note on the *Promptorium*, "Elfe, sprite. *Lamia*," which the compiler took from the *Catholicon* or *Summa* of John of Geneva, A.D. 1286, Mr. Way says:

The Catholicon explains *lamia* to be a creature with a human face, and the body of a beast, or, according to a gloss on Isai. xxxiv, 14, a sort of female centaur, which entered houses when the doors were closed, as old wives' tales went, and cruelly used the children, whence the name, "*quasi lania, a laniando pueros.*" The ancient leeches have given in their books numerous charms and nostrums for the relief of children "taken with elvys;" among which may be cited the following from a curions medical MS. of XVth cent. in the possession of Sir Thomas Phillipps. "For a chylde that ys elfe y-take, and may nat broke hys mete, that hys mouthe ys donne (*sic.*) Sey iij tymes thys verse, *Beata mater munere, &c.* In the worchyppe of God, and of our Ladi, sey iij pater noster, and iij aueys, and a crede; and he schal be hole." In Sloane MS. 73, f. 125, it is directed to "take þe roote of gladen and make poudre þereof, and ȝeue þe sike boþe in his metes, and in hise drynkis, and he schal be hool wiþinne ix dayes and ix nyȝtis, or be deed, for certeyn." William Langham, practitioner in physic, recommends this same remedy in his Garden of Health, 1579; and orders the root and seeds of the peony to be hung about children's necks, as a charm against the haunting of the fairies and goblins. The term elf is not, however, applied exclusively to mischievous spirits, but to fairies generally. See in Brand's Popular Antiquities detailed observations on the Fairy Mythology. "An elfe, *lamia, eumenis, dicta ab eu, quod est bonum, et mene, defectus.* Elfe lande," (no Latin word) CATH. ANG. Horman seems to speak of elves as a sort of vampires: "No man stryueth with deed men but elfis, *laruæ;*" and Palsgrave gives "elfe, or dwarfe, *nain.*" Ang.-Sax. elf, *lamia.*

On the *Catholicon Anglicum* Latin explanation of "Elfe," quoted by Mr. Way, there is the following note given by Mr. Herrtage:

'*Lamia.* A beaste that hath a woman's face, and feete of an horse.' Cooper. '*Satirus.* An elfe or a mysshapyn man.' Medulla. In the Man of Lawe's Tale, 754, the forged letter is represented as stating that

'the queen deliuered was
Of so horrible a feendly creature . . . .
The moder was an *elf*, by auenture
Ycome, by charmes or by sorcerye;'

and in the Chanoun's Yemannes Tale, 842, Alchemy is termed an '*eluish* lore.' Horman says: 'The fayre hath chaunged my chylde. *Strix, vel lamia pro meo suum paruulum, supposuit.*' In Aelfric's Glossary, Wright's Vol. of Vocab. p. 60, we have *elf* used as equivalent to the classical *nymph:* thus we find '*Oreades*, munt-ælfen; *Dryades*, wudu-elfen; *Hamadryades*, wylde-elfen; *Naiades*, see-elfen; *Castalides*, dun-elfen.' '*Pumilus.* An elfe or dwarfe.' Stanbridge, *Vocabula.*

In *Leechdoms, Wortcunning, and Starcraft of Early England*, edited by the Rev. Oswald Cockayne in the Rolls Series, will be found many recipes to be used for the physical evils wrought by elves. Halliwell and Wright in their additions to Nares's *Glossary* give "Elf-cake" as "an affection of the side, supposed, no doubt, to be produced by the agency of the fairies," and quote from *Lupton's Thousand Notable Things*: "To help the hardness of the side, call'd the *elf-cake*—Take the root of gladen, make powder thereof, and give the diseased party half a spoonful to drink in white-wine; or let him eat thereof so much in his potage at a time, and it will help him."

Some have thought that a reference to plica polonica lies in the words of Mercutio (*Romeo & Juliet*, I. iv. 90) in which he attributes to Mab the power of producing elf-locks. But, as Nares says, this is not probable. It will be remembered that in *Lear* (II. iii. 10) Edgar says that part of his disguise is to elf all his hair in knots.

